# Gender mainstreaming as a pathway for sustainable arbovirus control in Latin America

**DOI:** 10.1371/journal.pntd.0007954

**Published:** 2020-02-27

**Authors:** Clare Wenham, João Nunes, Gustavo Correa Matta, Carolina de Oliveira Nogueira, Polyana Aparecida Valente, Denise Nacif Pimenta

**Affiliations:** 1 Department of Health Policy, London School of Economics, London, United Kingdom; 2 Department of Politics, University of York, York, United Kingdom; 3 National School of Public Health, Fundação Oswaldo Cruz, Manguinhos, Rio de Janeiro, Brazil; 4 Instituto Rene Rachou–Fiocruz Minas, Fundação Oswaldo Cruz, Belo Horizonte, Minas Gerais, Brazil; 5 Universidade do Estado de Minas Gerais/Ibirité, Vila Rosário, Minas Gerais, Brazil; Centro de Pesquisa Gonçalo Moniz-FIOCRUZ/BA, BRAZIL

The 2015 to 2017 outbreak of Zika generated global attention on the risk of a spectrum of neurological disorders posed to women and their unborn children—including, but not limited to, microcephaly—that came to be known as congenital Zika syndrome (CZS). Images of women cradling babies born with CZS underscored the gendered nature of the epidemic. Nonetheless, the media attention towards the highly gendered dimensions of the outbreak was not matched by a recognition of the importance of female participation in the decision-making for the control of the *Aedes aegypti* mosquito, the vector responsible for the spread of Zika. Moreover, while women were the target population of the public health response to the epidemic, the impact of arbovirus policies on women was largely neglected.

This paradox—the absence of gender in the policy response to a problem where the gender dimensions were evident from the start—adds to other questions about the sustainability of arbovirus control.

The Zika epidemic is but one element of a broader problem with arboviruses—including dengue fever, yellow fever, and chikungunya—which by and large remain neglected across Latin America (and much of the world). Dengue fever, spread by the same *A*. *aegypti* mosquito, has shown considerable growth across the continent in recent years [1]. For example, Brazil reported close to 1.5 million cases of the disease between 2014 and 2016 [2]. This is mirrored across Latin America, where there have been almost 700,000 reported cases so far in 2019 alone [3]. Similarly, the region is witnessing the highest rates of other diseases transmitted by *A*. *aegypti*. This includes yellow fever—particularly in Brazil [4] [5]—and chikungunya, which was only introduced to the hemisphere in 2013 and is now present in almost every country in the region, causing a significant morbidity burden [6].

Another question pertains to the complex history of arbovirus control in the region, which has demonstrated some notable, if only temporary, successes [7]. Recognition of this history and of the historical ecology of mosquitoes in the region is essential for the effectiveness of present programs, which thus far have repeated the mistakes of the past. Brazil has eliminated *A aegypt*i numerous times [8] [9]. Nonetheless, the preference for vertical programs focusing on the “war” against *Aedes* has led to short-lived results, with mosquitoes returning within years, due, in a large part, to the absence of a coordinated regional response and the failure to consider and integrate the socioeconomic and structural determinants that enable mosquitoes to thrive. These include substandard living conditions, including those that result from rapid urbanization, increasing population density, poor quality housing, and inadequate sanitary and health facilities, along with lasting public sector deficiencies such as lack of routine water provision, which means that water must be collected and stored within houses for everyday use, providing a breeding ground for mosquitoes [7] [9]. Increasing resistance to pesticides has also been identified as a major and important obstacle [10]. Brazil has a rich history of involvement of civil, religious, and political movements in the health sector, particularly in advocacy for increased health provision, culminating in the 1970s in what came to be known as the collective health movement (saúde coletiva) [11] [12] [13]. In the wake of the Zika outbreak, mothers with children affected by CZS formed associations to fight for their children’s right to health. This echoes previous community-led campaigns. Thirty years earlier, during the first large dengue epidemic in Nova Iguaçu, Rio de Janeiro, women from this and other municipalities formed organized groups to campaign in the streets for the right to health, in an environment where unregulated urban growth, poor infrastructure, dependence on clientelism to finance healthcare and/or prevention mechanisms, and the scourge of violence had led to dengue infecting hundreds of new victims daily [14]. This is another reason for a systematic consideration of the history of past interventions.

If an awareness of history is important when thinking about sustainable arbovirus control, so is the recognition of contemporary context, which in recent years has been volatile. One notable example is the political turmoil in Venezuela, which has led to a rapid increase of arboviruses within the country, with dengue incidence increasing 4-fold and chikungunya cases reaching 2 million in the country since 2014 [15]. Due to the exodus of Venezuelans into neighboring countries, the incidence of these diseases is spilling over into the region and posing a major public health crisis, as neighboring countries struggle to limit the soaring rates of disease among displaced communities and beyond.

The sustainability of the public health response was also impacted by the framing of Zika as a health emergency [16]. A “grammar of security” [17] was deployed by actors at subnational, national, regional, and global levels, and extraordinary measures were adopted as a consequence, including fumigation and military and civic involvement in vector control [18, 19]. The security framing contributed to vector control assuming center stage but at the same time led to a narrow and short-term approach focused on containment and crisis management. Discussions about economic and social determinants, access to health information, and reproductive, disability, and maternal rights were largely marginalized from public policy considerations. Women were instructed by governments to avoid getting pregnant and to avoid mosquito bites if pregnant. Such recommendations were not accompanied by adequate provision of healthcare information and services to allow for informed decisions about their health, particularly reproductive health or the health of their unborn child. They also neglected the socioeconomic context in which individual choices are made.

Against the background of scientific uncertainties surrounding CZS and the instability in Zika’s aetiology, transmission, and treatment [20], recommendations added to a gendered context of violence, stigma, and restrictive reproductive health options, such as the criminalization of abortion and a lack of access to contraceptives [21] [22]. Importantly, despite the fact that the key group affected by the Zika outbreak was women of childbearing age, control programs failed to meaningfully engage with these or other women. For example, in Brazil the key public health campaign targeted health workers rather than the women themselves, leaving them systematically excluded from the outbreak response [23]. Similarly, women were largely absent from the decision-making and from the implementation of public health policies at the regional and global levels [24].

We call for a paradigm shift in arbovirus control in specific and vector control in general. Sustainable arbovirus control needs to go beyond short-term measures and engage in a serious manner with the historical and socioeconomic context of interventions. Gender is paramount in this context. Women and gender considerations have been absent from planning for arbovirus control, both in policy and in academic discussions, and this presents a problem for the sustainability of these interventions. We thus make the case for mainstreaming gender considerations, mainstreaming being defined as “the efforts to scrutinize and reinvent processes of policy formulation and implementation … to address and rectify persistent and emerging disparities between men and women” [25].

This viewpoint lays out a future research agenda to address the following questions: why *Aedes* control hasn’t worked and what the role of gender in this failure is; whether a gender-mainstreamed vector control program may offer more sustainability for infectious disease management; and whether women’s meaningful involvement in arbovirus control may contribute to broader societal gender equality.

## The importance of gender in vector control

There is widespread consensus that vector control programs, including those for arboviruses, must be context specific and that their success hinges upon meaningfully engaging with local social, economic, and cultural factors [26] [27] [28]. However, gender is often neglected as a contextual factor for such disease control efforts. Several gendered dimensions require consideration.

To begin with, gender plays a crucial role as a determinant of disease exposure and vulnerability. Because of existing societal gender norms, women are traditionally responsible for care and work in the household and water collection and storage, in addition to a significant portion of agricultural work. These activities put them in closer contact with mosquito breeding sites and therefore at greater risk of contracting arboviral diseases [29] [30]. As the Zika epidemic has further demonstrated, gender structures also influence people’s ability to deal with or alleviate the short-, medium-, and long-term effects of disease, including access to the full range of health services they are entitled to—in particular, reproductive health provision. Zika also underscores how disease can deepen gender-based vulnerabilities. It is symptomatic of existing patriarchal structures in many Latin American countries that Zika heightened the burden faced by women. For example, for many women, traditional care responsibilities have extended to palliative support for children with CZS, compounded by the fact that in many cases they were abandoned by their partner after a CZS diagnosis.

Relatedly, and as highlighted by Nading’s work on Nicaragua [31], the role played by women in vector control programs reflects broader dynamics in the distribution of labor in global healthcare, with women dominating low (or un-) paid roles in the frontline implementation of these programs. As Nading highlights, “in practice, if not by design,” women end up being the primary deliverers of vector control strategies [31]. At the same time, there’s little representation of women amongst higher-ranking positions of power, decision-making, or policy design. In addition to this “glass ceiling” in the design of vector control, policies have not been designed to account for the increased burden of vector control activities on female health workers, upon whom society already places high expectations of care (for their families and their communities). The Zika epidemic has generally meant longer working hours without a reduction of other responsibilities or an increase in incentives or salary. Overall, the use of women in these programs may be said to correspond to a feminization of labor that reproduces and can even heighten existing inequalities [32]

The involvement of women in the implementation of vector control reflects societal perceptions of gender roles. In Brazil, for example, community health workers (agentes comunitários de saúde) are part of the primary health system. They are overwhelmingly women and tasked with disease prevention and health promotion activities, which in the case of Zika focused on altering individual behaviors and identifying mosquito hot spots. At the same time, vector control agents (agentes de combate a endemias) work alongside the community health workers on the ground. These are predominantly male and, contrastingly, from the epidemiology division tasked with surveillance and control activities, such as diminishing vector breeding sites and mechanical or chemical control of mosquitoes. This gendered division of labor speaks to a vision of primary healthcare as being “feminine” and of household visits by community health workers as “maternal” interventions relying on skills traditionally associated with women (such as empathy and persuasion). This is in contrast with more “masculine” interventions relying on technological and pharmacological instruments but also on sheer physical prowess (for example, the ability to climb onto people’s roofs in order to administer larvicide in water tanks). This dovetails with research on the role of gender in vector control programs in Africa and Southeast Asia, which suggested that men are more likely to be involved in programs comprising physical labor such as fumigation and improvements to sanitation, while women are more likely to be involved in community education programs, which have more long-term effects [33] [34].

Vector control programs also tend to perceive women as an unproblematic “target group.” In the context of neglected tropical diseases and vector control policies, women, particularly from low and middle income countries, are commonly thought of and labeled as a homogenous group, independent of class, race, social status, age, locality, and other social markers. Feminist analysis and science and technology studies (STS) have long recognized that assuming a universal female experience is deeply problematic [35]. The category “woman” is not defined by any common biological or psychological characteristics; rather, individuals are positioned as women by a set of material and immaterial social constructs. There is also great diversity of experiences along different social markers and within them, intersections that need to be considered [36]. Thinking of women as a series or a social collective acknowledges that individuals exist within structures that constrain and channel their actions in particular ways [37]. Recognizing diversity is essential for designing policies that are cognizant of interlocking vulnerabilities affecting women’s lives and choices, which can only be grasped by engaging with local context and concrete experiences. In addition to potentially contributing to the effectiveness and sustainability of these programs, this awareness of heterogeneity also helps to prevent programs that inadvertently reproduce existing inequalities and injustices.

As is well known, the proliferation of mosquitoes is inherently tied to the inadequate provision of water and sanitation infrastructure and garbage disposal. Infrastructural deficiencies, in turn, raise broader questions regarding neoliberal reforms in Latin American states and the long-term impact of the hollowed state activity to manage vector control and deliver public services more generally. Research has shown the disproportional effects of neoliberal reforms on women [38–40]. Recognition of these gendered effects in vector control strategies is essential for ensuring their context sensitivity and ultimately their success over time. At the same time, however, vector control will not be able to address systemic inequalities by itself. Public health interventions need to be accompanied by broader efforts of political change led by women themselves. Recent examples of social mobilization around infrastructural determinants [41] offer clues on how to conjoin struggles for health, quality of life, and justice.

In sum, gender mainstreaming, as a strategy for placing these gendered dimensions at the center of attention, can decisively contribute to the effectiveness and long-term sustainability of arbovirus control. Previous studies of mosquito control in Sudan, Kenya, and Indonesia have shown that women are more likely to create self-sustaining vector control programs [33] and that such programs can contribute towards broader gender equity [42]. This may in part relate to local division of labor but also points to societal power changes that may result from placing women at the helm. As authors from a study of 20 years of gender mainstreaming in health have acknowledged, gender intersects with other axes of inequity such as ethnicity, socioeconomic status, occupation status, age, sexuality, (dis)ability, and religion [43]. These intersections offer a wealth of possibilities for mainstreaming gender in arbovirus control in a sophisticated way. Intersectionality analysis is thus key to addressing this global health issue. Importantly, mainstreaming gender into vector control policies will necessarily involve addressing gender equality more broadly. This will entail a broader discussion about women’s rights, including access to contraception and the decriminalization of abortion, which are still thorny issues in the region.

## Engendering research and health policies

Much remains to be understood about the role of gender in arbovirus prevention and control policies. A recent systematic analysis concluded that there had not been significant research undertaken to understand the relative effectiveness of women-centered vector control programs [44]. There has been no study of how traditional gender divisions of labor impede sustainable *Aedes* control and of the potential implications of changing vector control activity in terms of addressing inequality and enhancing the agency of women. Moreover, the current literature focuses on vector control more broadly, and there have not been comprehensive studies on how gender affects arbovirus control programs in particular.

Therefore, we call for more research on the various gender aspects of arbovirus control. This includes, but is not limited to, studies on the following: women’s participation in arbovirus control programs, the nature of this participation, and the extent to which it might result in improved outcomes; the impact of *Aedes* control programs on women and how sustainable these programs have been in limiting the spread of disease; the historical impact of women participation in arbovirus control, including women who produce, analyze, and implement evidence in science, policy, and managerial activities; the long-term effectiveness of programs; the division of labor in the design and implementation of policies and their underlying assumptions regarding gender roles and the place or women in society; the heterogeneity of women experiences and the intersection of gender with other potential sources of vulnerability such as class or race; the relationship between women, territories, living spaces, and infrastructure; and the potential for broader political transformation, in terms of gender equity and equality, through gender mainstreaming in health policy and health-related activism. Building on the UN’s Sustainable Development Goals commitment to “leave no one behind” and the recent championing of a need for a people-centered approach to epidemic preparedness and response [45], we call for more evidence that can support policy recommendations and advocacy efforts for gender-mainstreamed arbovirus control programs in Latin America.
